# Battery Pack for IoT Devices in a Harsh Outdoor Environment

**DOI:** 10.3390/s26165232

**Published:** 2026-08-18

**Authors:** Peter Ševčík, Michal Hodoň, Lukáš Formanek, Peter Šarafín

**Affiliations:** Department of Technical Cybernetics, Faculty of Management Science and Informatics, University of Žilina, Univerzitná 8215/1, 010 26 Žilina, Slovakia; michal.hodon@fri.uniza.sk (M.H.); lukas.formanek@fri.uniza.sk (L.F.); peter.sarafin@fri.uniza.sk (P.Š.)

**Keywords:** IoT, battery pack, LiFePO_4_, fuel gauge, protection circuit, low-temperature operation, outdoor sensor node

## Abstract

Outdoor Internet of Things (IoT) sensor nodes require battery systems for which their behaviour and implementation limits are characterized under low-temperature and variableload conditions. This study documents a LiFePO_4_ battery-pack prototype integrating a BQ29729DSET protection IC and a configured MAX17055ETB+T fuel gauge and descriptively compares its discharge runtime with that of a reference pack with a similar nominal capacity, comprising three parallel Samsung ICR18650-26H cells. Tests were conducted at −30 °C, 8 °C, and 25 °C under nominal load settings of 50, 100, and 200 mA. At 8 °C and 25 °C, the two configurations showed similar runtimes and nominal-current-based calculated capacities. At −30 °C, the LiFePO_4_ assembly ran for 89.1 versus 66.0 h at 50 mA and 44.7 versus 37.2 h at 100 mA. An analysis based on the typical MCP1700 dropout characteristic bounds the portions of these LiFePO_4_ runtimes recorded below the assumed regulation threshold at approximately 1.1 h and 0.9 h, respectively; even subtracting those complete intervals leaves positive differences of 33.3% and 17.7% relative to the reference runtimes. Complete current logs were unavailable; therefore, capacity and energy are reported only as nominal-current estimates. In the ICR18650-26H reference pack at −30 °C, the calculated capacity increased anomalously from 3302 to 4087 mAh as the nominal setting increased from 50 to 200 mA. The ICR cutoff remained above the estimated regulator-dropout thresholds, so dropout does not explain the anomaly; temperature, conditioning, and run-order effects cannot be excluded. Protection trip points and fuel-gauge accuracy were not experimentally verified. Our contribution is therefore reproducible design documentation combined with preliminary low-temperature runtime evidence rather than validation of a fully monitored and protected battery pack.

## 1. Introduction

Battery-powered Internet of Things nodes are increasingly deployed in outdoor and industrial locations where wired power is unavailable and maintenance access is limited. In these applications, the power subsystem is a reliability-critical part of the node: low temperatures, communication-related current peaks, long sleep intervals, and ageing can reduce usable capacity and terminal voltage and can impair estimates of the remaining operating time.

Recent research addresses several parts of this problem. Hasan et al. compared primary and secondary battery technologies for different IoT applications and showed that battery selection must be application-specific rather than based on energy density alone [1]. At the device and network levels, stochastic depletion models [2], protection against battery-draining traffic [3], and reduced idle-listening energy [4] improve lifetime prediction or reduce demand. Energy-harvesting reviews and implemented platforms extend autonomy by combining ambient sources with storage and energy-aware scheduling [5,6,7,8]. These contributions are important, but they primarily address chemistry selection, energy demand, or harvested-energy availability rather than the implementation and environmental evaluation of the rechargeable battery pack itself.

Battery-state monitoring constitutes a second research stream. Data-driven methods can estimate state of charge from voltage, current, and temperature measurements [9], while IoT-connected battery-management prototypes can monitor pack parameters and cell balancing [10]. For outdoor nodes, meteorological variables have also been used to predict the battery level of solar-powered devices and adapt their sampling rate [11]. However, an accurate estimator or cloud-monitoring layer does not replace independent hardware fault protection, and studies performed at a fixed environmental temperature do not establish discharge behaviour under severe cold conditions.

Temperature-specific battery studies provide the third part of the background. Measurements on a commercial LiFePO_4_ module over −20 to +55 °C demonstrated a marked capacity reduction below room temperature and emphasized the need for configuration-specific thermal characterization [12]. At −30 °C, an actively heated Li-ion pack with carbon-nanotube sheets recovered much of its room-temperature capacity, but it required a heater, feedback control, and additional energy [13]. These studies establish both the effect of temperature and the passive-versus-active thermal-management trade-off, but they do not evaluate the same combination of a low-power IoT-oriented source, independent protection, host-accessible fuel gauge, and discharge-data acquisition used here.

The resulting research gap is therefore an integration and documentation/evidence gap, not the absence of individual battery, protection, or monitoring technologies. The literature provides limited experimental evidence for compact outdoor-IoT battery packs in which (i) cell selection, (ii) host-independent fault protection, (iii) fuel-gauge configuration, and (iv) temperature-dependent discharge behaviour are documented together. Section 2 provides a structured comparison of the closest research streams and their boundaries.

This work addresses the gap through the design and discharge-side evaluation of a compact LiFePO_4_ battery pack prototype. The architecture combines a Headway HW 38120HP cell (Zhejiang Xinghai Energy Technology Co., Ltd., Huzhou, China), a BQ29729DSET hardware protection stage (Texas Instruments, Dallas, TX, USA) designed to operate independently of the host microcontroller, and a MAX17055ETB+T fuel gauge (Analog Devices, Wilmington, MA, USA). The experimental comparison uses a Samsung ICR18650-26H reference pack (Samsung SDI Co., Ltd., Yongin, Republic of Korea) with a similar nominal capacity, comprising three parallel cells, at three ambient temperatures and three nominal load currents. Because the configurations differ in cell format, cell count, nominal energy, thermal mass, internal resistance, and rated current capability, all comparisons are made at the assembled power-source level rather than interpreted as a chemistry-wide ranking.

This study has three explicit research objectives. **O1** is to design and reproducibly document a compact sensor-node power architecture that combines a single-cell LiFePO_4_ source, host-independent fault-protection hardware, and host-accessible battery-state-monitoring hardware. **O2** is to describe the discharge-side behaviour of the proposed and reference assemblies across the full 2 × 3 × 3 configuration–temperature–nominal-load design using measured time to cutoff, calculated capacity and energy estimates, average discharge voltage, and temperature-normalized retention. **O3** is to determine which conclusions are supported by the surviving discharge-plot images and the curves digitized from them and to identify the measurements still required for functional and field qualification.

Because the experiment contains one assembly and one record per condition, a formal inferential hypothesis is not appropriate and the earlier voltage-profile hypothesis has been withdrawn. The descriptive research question is **RQ1**: At −30 °C, how does measured time to the configuration-specific cutoff differ between the HW 38120HP and ICR18650-26H assemblies under the same 50 and 100 mA nominal load settings? The 200 mA setting is treated as a higher-load diagnostic condition. RQ1 does not assume constant measured current, a stable voltage plateau, chemistry-wide superiority, or statistical significance.

The technical contribution claimed in this paper is deliberately limited: requirement-driven and reproducible design documentation for protection and monitoring hardware within an outdoor-IoT sensor-node power subsystem, accompanied by 18 preliminary discharge records and an explicit account of their anomalies and validation boundaries. The discharge experiment does not validate protection operation or fuel-gauge accuracy. No claim is made that the chemistry, protection IC, fuel-gauge IC, or analytical method is novel.

## 2. Related Work and Battery-Pack Design Context

The power source of an outdoor IoT node must be selected according to the electrical load profile, required maintenance interval, operating-temperature range, safety requirements, physical constraints, and expected service life. Relevant battery parameters include nominal and usable capacity, nominal voltage, energy density, internal resistance, cycle life, maximum charge and discharge currents, and manufacturer-specified temperature limits. At the system level, battery selection must also consider the minimum operating voltage of the downstream electronics and the current peaks associated with sensing, processing, and wireless communication [14,15].

Conventional lithium-ion cells are widely used in portable and embedded systems because of their relatively high gravimetric and volumetric energy density. However, they require protection against overcharge, excessive discharge, overcurrent, short-circuit conditions, and operation outside the permitted temperature range [16,17,18]. The Samsung ICR18650-26H cell used in the reference configuration has a nominal voltage of approximately 3.63 V, a nominal capacity of 2600 mAh, and a manufacturer-specified end-of-discharge voltage of 2.75 V [19].

Lithium iron phosphate cells generally have a lower nominal voltage and lower specific energy than high-energy lithium-ion cells, but they are frequently considered for applications in which thermal stability, cycle life, and predictable discharge-voltage behaviour are prioritized. The Headway HW 38120HP cell evaluated in this work has a nominal voltage of 3.2 V, a nominal capacity of 8 Ah, a maximum charging voltage of 3.65±0.05 V, and a specified end-of-discharge voltage of approximately 2.5 V [20]. The difference between the voltage ranges of the two cell types affects the selection and configuration of the monitoring, protection, and downstream power-conversion circuits.

Battery monitoring can improve system autonomy by allowing the host device to modify its sampling interval, communication frequency, or shutdown strategy according to the estimated remaining energy. Fuel-gauge ICs combine measurements of voltage, current, and temperature with battery models and coulomb counting to estimate state of charge, remaining capacity, and runtime. The MAX17055ETB+T selected for the proposed pack supports single-cell lithium-ion variants, including LiFePO_4_, and provides battery-state information through an I^2^C interface [21]. Because the open-circuit-voltage profile of LiFePO_4_ cells is relatively flat over a substantial state of charge interval, the accuracy of the estimate depends on appropriate gauge configuration and, where required, cell-specific characterization.

An independent protection circuit is also required because battery safety must not depend solely on correct host-microcontroller operation. Protection ICs detect abnormal voltage and current conditions and control external switching devices to isolate the cell from the load or charging path. The BQ29729DSET datasheet specifies hardware-level undervoltage, overvoltage, overcurrent, and short-circuit detection [22]. Its detailed thresholds and their compatibility with the selected LiFePO_4_ cell are discussed in Section 4.2.

### 2.1. Structured Comparison of the State of the Art

To identify the system-level gap, recent work was classified according to the function evaluated: battery selection, device-level energy demand, energy harvesting, battery-state estimation, IoT-connected pack monitoring, outdoor battery prediction, or low-temperature cell performance. Table 1 compares the evidence provided by each stream with the four functions required in this study: an implemented rechargeable pack, independent protection, host-accessible state monitoring, and controlled low-temperature discharge evaluation. A dash indicates that the cited study did not evaluate the corresponding function; it does not imply a deficiency relative to that study’s stated objective.

### 2.2. Characteristics of the Tested Power-Source Configurations

The discharge experiment compared two commercial power-source configurations. The first configuration consisted of three Samsung ICR18650-26H cells connected in parallel, providing a nominal capacity of approximately 7.8 Ah. The second configuration consisted of one Headway HW 38120HP LiFePO_4_ cell with a nominal capacity of 8 Ah. The configurations were therefore selected to provide similar nominal capacities. The main features of the compared battery packs are summarized in Table 2.

The configurations were not identical in nominal energy, physical size, cell count, thermal mass, internal resistance, or rated current capability. The nominal energy of the three-cell ICR18650-26H parallel pack is approximately 28.3 Wh, whereas that of the HW 38120HP cell is approximately 25.6 Wh. These differences are considered in the interpretation of the discharge results.

### 2.3. Research Gap

The comparison in Table 1 shows that current studies generally optimize one layer of the problem: chemistry selection, energy consumption, harvesting, estimation, connected monitoring, or temperature-dependent cell behaviour. Relatively little attention has been given to compact outdoor-IoT battery packs in which cell selection, independent hardware-protection design, fuel-gauge configuration, and temperature-dependent discharge behaviour are documented together. This work addresses this documentation gap through a LiFePO_4_ prototype and a discharge-side runtime assessment. The claimed contribution is a reproducible architecture description and a boundary-aware account of the surviving measurements, not functional validations of protection or monitoring and not a new electrochemical material, protection algorithm, or state-estimation algorithm.

## 3. Design Requirements, Evaluation Criteria, and Technical Constraints

The target application is an autonomous outdoor IoT sensor node intended for operation in environments with limited maintenance access. The battery pack must support low-power sensing and communication cycles, provide usable voltage under the expected temperature and load conditions, detect excessive discharge and fault currents independently of the host, and communicate battery-state information to the host device. The present study focuses on discharge behaviour, fuel-gauge integration, and protection architecture. Charging-circuit design and charge-side experimental validation are outside its scope. The requirement table below distinguishes design targets from the evidence obtained in the present study so that an implemented function is not inadvertently described as experimentally verified.

### 3.1. Alignment of Objectives, Research Question, and Evaluation

Table 3 defines the method and interpretation criterion associated with each objective and RQ1. O1 is evaluated primarily by circuit- and configuration-level analyses; O2 and RQ1 use the discharge records; O3 combines those two evidence streams with a state-of-the-art comparison. This mapping is maintained in the Results, Discussion, and Conclusions Sections.

### 3.2. Electrical and Environmental Requirements

The BQ29729DSET thresholds listed in Table 4 are fixed properties of the selected component, not application requirements reconstructed after component selection. Its 4.275 V overvoltage threshold is above the normal maximum voltage of the selected LiFePO_4_ cell and is therefore treated as secondary abnormal-condition protection rather than as chemistry-specific charge termination. Normal charge-voltage control must be provided separately by the charging system, which is outside the scope of the present study.

The MAX17055ETB+T provides battery-state information through the I^2^C interface. Its cell capacity, empty-voltage threshold, charge-termination current, sense-resistor scaling, and battery model must correspond to the implemented cell and circuit. The configuration used in the prototype is described in Section 4.3.

### 3.3. Capacity, Energy, and Runtime Estimation

When complete current and voltage records are available, delivered capacity and energy can be calculated by time integration. With time expressed in seconds, the delivered capacity in ampere-hours is

(1) $$Q_{\mathrm{Ah}}=\frac{1}{3600}\int_{0}^{t_{\mathrm{end}}}I\left(t\right)\;dt,$$

and the delivered energy in watt-hours is

(2) $$E_{\mathrm{Wh}}=\frac{1}{3600}\int_{0}^{t_{\mathrm{end}}}V\left(t\right)I\left(t\right),dt,$$

where I(t) is the measured discharge current, V(t) is the measured terminal voltage, and $t_{\mathrm{end}}$ is the time at which the defined cutoff condition is reached.

For discrete samples with timestamps expressed in seconds, the corresponding numerical estimates are

(3) $$Q_{\mathrm{Ah}}\approx \frac{1}{3600}\sum_{k=1}^{N-1}I_{k}\Delta t_{k},\;E_{\mathrm{Wh}}\approx \frac{1}{3600}\sum_{k=1}^{N-1}V_{k}I_{k}\Delta t_{k},$$

where $\Delta t_{k}=t_{k+1}-t_{k}$. Using the individual sampling intervals allows the calculations to remain valid when the data-logger interval is not perfectly constant.

Complete INA219 current records were not available for the result set analyzed in this manuscript. The reported values were therefore calculated from the nominal current $I_{\mathrm{nom}}$, the measured runtime, and the digitized voltage–time curves:

(4) $$Q_{\mathrm{calc},\mathrm{Ah}}=\frac{I_{\mathrm{nom}}t_{\mathrm{end}}}{3600},\;E_{\mathrm{calc},\mathrm{Wh}}\approx \frac{I_{\mathrm{nom}}}{3600}\sum_{k=1}^{N-1}V_{k}\Delta t_{k}.$$

Throughout this manuscript, calculated capacityand calculated energy mean the nominal-current estimates in Equation (4); these terms are used consistently and are not synonyms for measured-current-integrated quantities. The estimates are time-based proxies. Regulator dropout, resistor tolerance, temperature, and component self-heating can cause the actual current to differ from $I_{\mathrm{nom}}$, and the resulting bias need not be equal across temperature or load settings.

Capacity retention at temperature *T* is calculated relative to the corresponding result at 25 °C for the same power-source configuration and nominal load current:

(5) $$R_{Q,\mathrm{calc}}\left(T,I\right)=\frac{Q_{\mathrm{calc}}\left(T,I\right)}{Q_{\mathrm{calc}}\left(25\;{}^{\circ}C,I\right)}\;\times \;100$$

The measured terminal voltage is interpreted using a simplified equivalent-circuit relationship

(6) $$V_{t}\left(t\right)=V_{\mathrm{OCV}}\left(\mathrm{SoC}(t),T\right)-I\left(t\right)R_{0}\left(T\right)-V_{p}\left(t\right),$$

where $V_{\mathrm{OCV}}$ is the open-circuit voltage, $R_{0}$ represents the instantaneous ohmic resistance, and $V_{p}$ represents slower polarization effects. The model is used qualitatively to interpret the initial voltage decrease following load connection, the central discharge plateau, and the steep terminal-voltage decrease near depletion.

### 3.4. Technical Constraints and Their Treatment

The interpretation of the experimental results must account for constraints associated with the selected components, measurement system, tested power-source configurations, and available sample size. These constraints and their treatment are summarized in Table 5.

## 4. Proposed Battery-Pack Architecture

Based on the requirements in Table 4, the Headway HW 38120HP LiFePO_4_ cell was selected for the proposed battery pack. These characteristics motivated its selection for an outdoor-IoT prototype in which predictable discharge behaviour, safety, and limited maintenance are design priorities.

The three-cell Samsung ICR18650-26H parallel pack was used only as an experimental reference configuration. Its nominal capacity was selected to be close to that of the HW 38120HP cell. It is not part of the proposed LiFePO_4_ battery-pack architecture. Differences between the tested configurations include cell chemistry, cell format, cell count, nominal energy, internal resistance, thermal mass, and rated current capability. These differences are considered when interpreting the discharge measurements.

### 4.1. Protection and Monitoring Architecture

**Figure 1:**
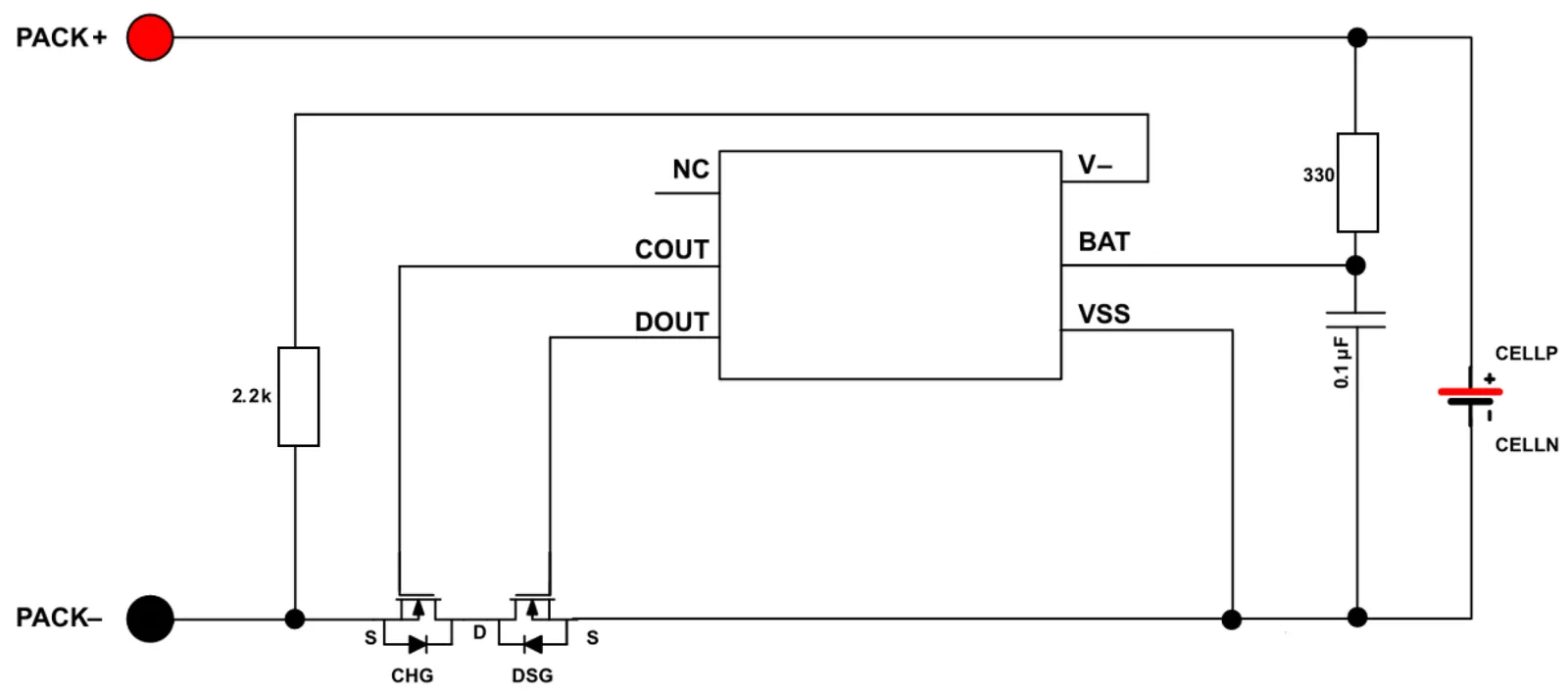
BQ29729DSET protection circuit implemented in the proposed battery pack. The circuit is designed for host-independent voltage- and current-fault detection according to the device’s specification [22].

**Figure 2:**
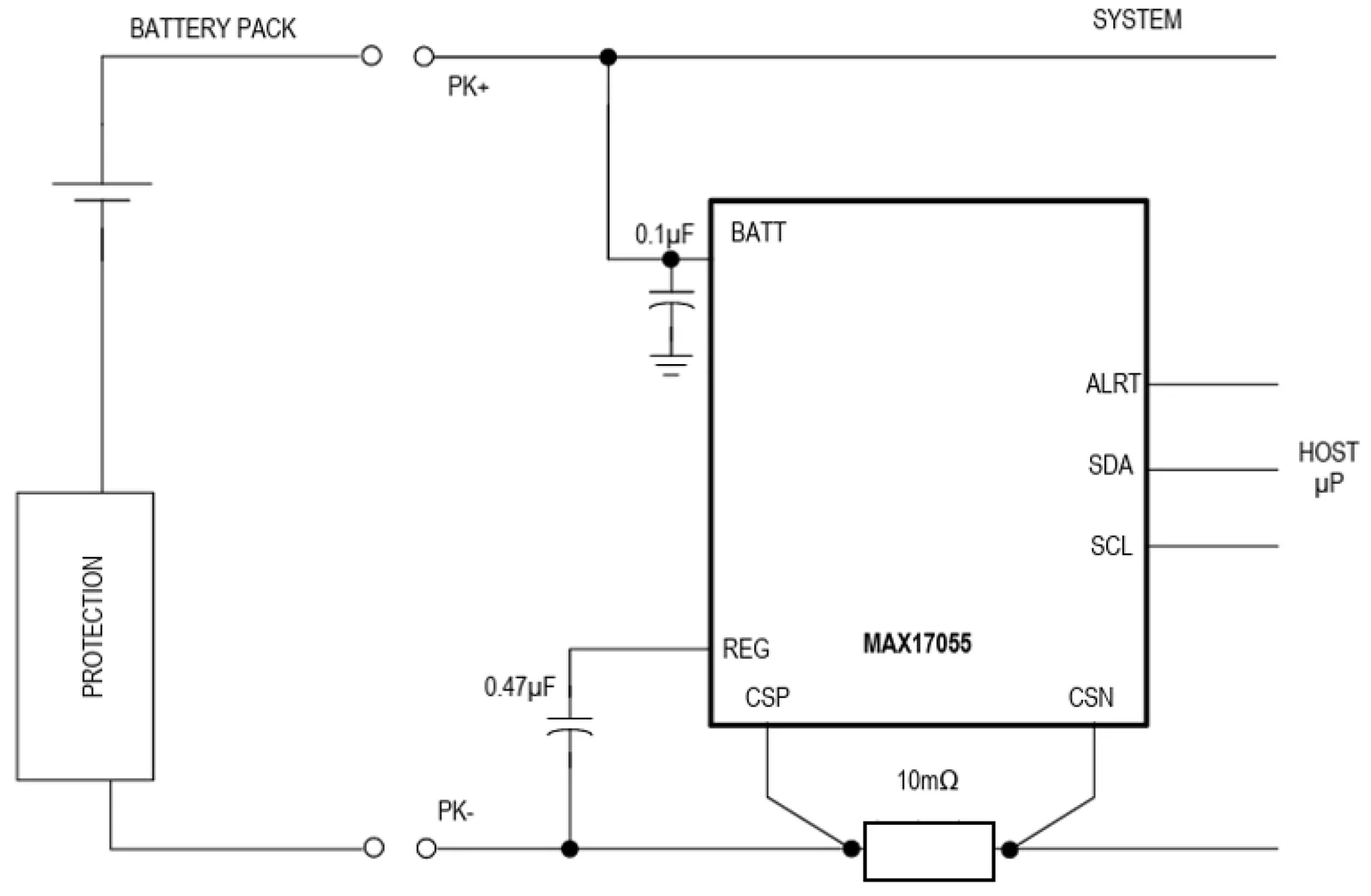
MAX17055ETB+T fuel-gauge circuit used for battery-voltage and current measurement and for state of charge, remaining-capacity, and runtime estimation [21].

**Figure 3:**
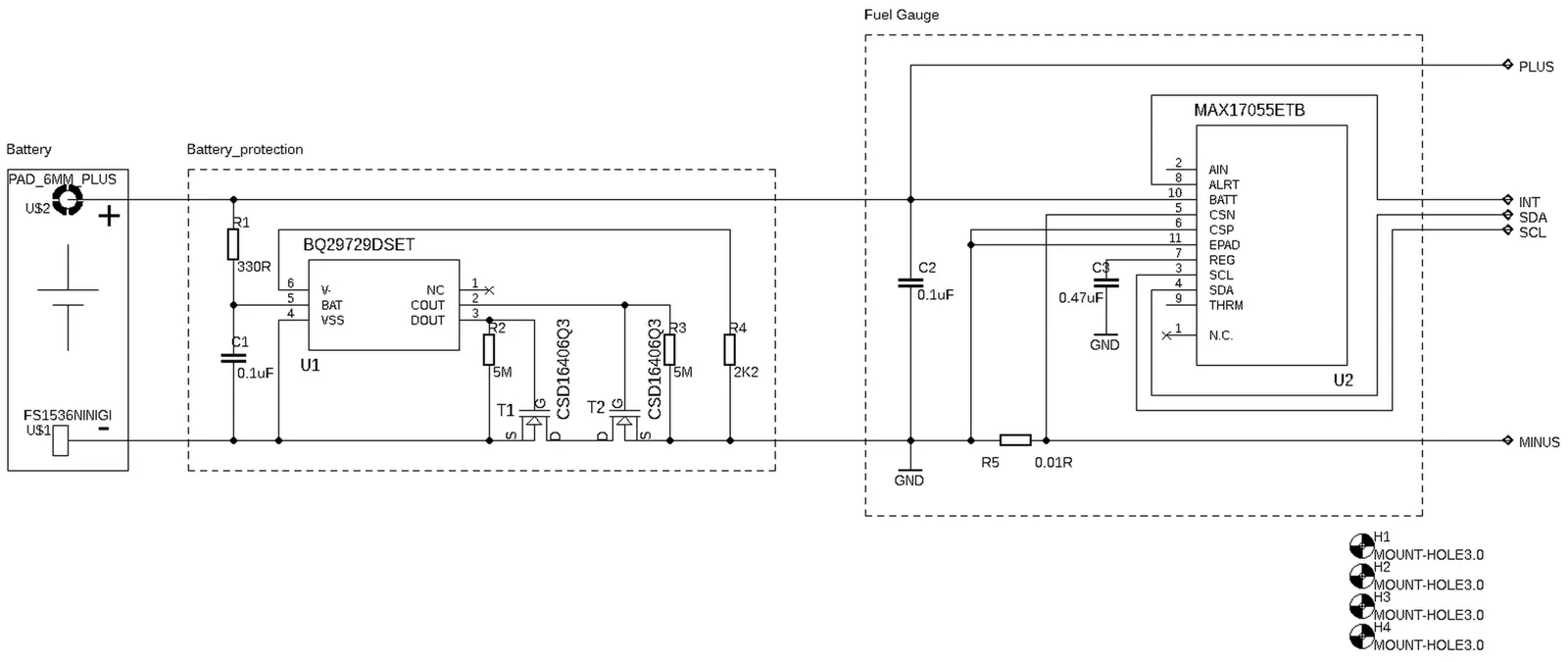
System schematic of the proposed battery pack, including the LiFePO_4_ cell, BQ29729DSET protection stage, MAX17055ETB+T fuel gauge, current-sense resistor, and external interface.

The proposed battery pack combines the HW 38120HP cell, a BQ29729DSET hardware protection stage, a MAX17055ETB+T fuel gauge, a 10 mΩ current-sense resistor, and an external power and communication interface. The protection stage is connected in a host-independent topology, whereas the fuel gauge is configured to provide battery-state information to the host through the I^2^C interface. The protection and monitoring circuits and the overall electrical interconnection are shown in Figure 1, Figure 2 and Figure 3.

The BQ29729DSET circuit implements hardware intended to detect cell undervoltage, overvoltage, discharge overcurrent, charge overcurrent, and short-circuit conditions. The MAX17055ETB+T is configured to measure battery voltage and current and to estimate state of charge, remaining capacity, and runtime-related quantities. Together, these devices are intended to provide independent fault detection and host-accessible battery-state monitoring.

### 4.2. BQ29729DSET Protection Strategy

BQ29729DSET was selected at the prototype-design stage because it supports a single-cell topology, external charge and discharge MOSFET control, host-independent voltage- and current-fault detection, and low-power operation. It was not selected because its fixed voltage thresholds are optimized for LiFePO_4_ chemistry. The compatibility analysis below shows that this choice is a design compromise: It documents an independent secondary isolation and current-fault architecture, but it cannot serve as the primary chemistry-matched overvoltage safeguard. A field revision should replace it with a LiFePO_4_-specific protection device for which its overvoltage threshold is coordinated with the 3.65 V charge limit.

The BQ29729DSET layer is designed not to depend on correct operation of the host firmware. This separation is important because the host microcontroller may reset, enter an undefined state, or become disconnected during field operation. The relay controlled by the ESP32-S3 measurement platform is used only to terminate laboratory discharge tests and is not treated as part of the autonomous battery-pack protection function.

The fixed BQ29729DSET protection thresholds are an overvoltage threshold of 4.275 V with a nominal delay of 1.25 s, an undervoltage threshold of 2.300 V with a nominal delay of 20 ms, a charge-overcurrent threshold of −0.100 V with a nominal delay of 8 ms, a discharge-overcurrent threshold of 0.130 V with a nominal delay of 8 ms, and a short-circuit threshold of 0.5 V with a nominal delay of 250 μs [22].

The 4.275 V overvoltage threshold is above the normal maximum charging voltage of the selected LiFePO_4_ cell. BQ29729DSET is therefore not used as the normal LiFePO_4_ charge-termination mechanism or as chemistry-specific charge-voltage control. Instead, its overvoltage function is treated as secondary protection against an abnormal fault condition. Normal charge-voltage regulation must be provided by the charging system, which is outside the scope of the present study.

The overcurrent and short-circuit values specified for the BQ29729DSET are voltage thresholds at the protection IC sensing input. The corresponding pack-current thresholds depend on the resistance of the external MOSFETs, PCB traces, interconnections, and other elements in the current path. Consequently, the actual trip currents, response delays, and recovery behaviour must be verified on the assembled protection circuit.

### 4.3. MAX17055ETB+T Fuel-Gauge Integration

MAX17055ETB+T is used to measure battery voltage and current and to provide state of charge, remaining-capacity, and runtime-related estimates to the host through the I^2^C interface. The pack current is measured using a 10 mΩ, ±1% sense resistor connected between the CSP and CSN pins.

With the 10 mΩ sense resistor, the DesignCap register was programmed for a nominal capacity of 8000 mAh, corresponding to 0x3E80. The IChgTerm parameter was set to 400 mA, corresponding to 0x0A00. Although charge-side validation is outside the scope of this study, IChgTerm is required by the fuel-gauge end-of-charge-detection algorithm.

The application empty-voltage target was set to 3.00 V and the empty-detection recovery voltage was set to 3.12 V, corresponding to VEmpty = 0x964E. Four voltage definitions are intentionally separated. First, approximately 2.5 V is the manufacturer-specified end-of-discharge voltage of the HW 38120HP cell. Second, 3.00 V is the MAX17055 application-level empty target; the gauge may therefore report zero state of charge while a reserve remains above the cell limit, and 3.12 V is its empty-detection recovery setting. Third, 2.5 V for the HW 38120HP assembly and 2.8 V for the ICR reference are measurement-bench termination thresholds used only to define $t_{\mathrm{end}}$. Fourth, 2.300 V is BQ29729DSET’s fixed undervoltage fault threshold. The fuel-gauge reference and the laboratory capacity endpoint are thus not interchangeable. Any future gauge-accuracy validation must define zero state of charge at the 3.00 V application threshold, whereas discharge-to-manufacturer-limit testing addresses a different quantity.

The standard ModelGauge m5 EZ LiFePO_4_ model was selected using ModelID = 6. No cell-specific custom characterization model was loaded. During initialization, the ModelCfg refresh command was issued using 0x8060. The SDA and SCL lines are pulled up to the 3.3 V logic supply through 2.2 kΩ resistors. The intended initialization sequence writes the configuration registers after a fuel-gauge power-on reset and then requests register readback; however, no synchronized readback records were available for the analyzed dataset. MAX17055 uses its internal temperature measurement.

**Figure 4:**
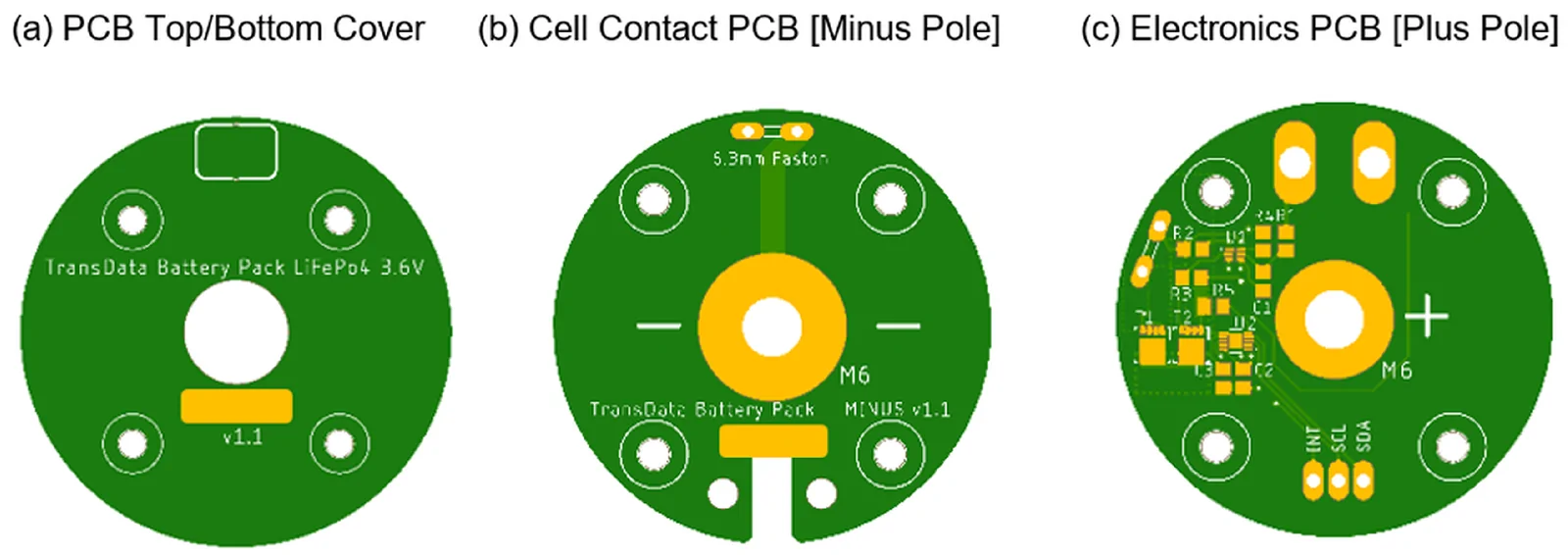
Circular PCB implementation of the battery-pack protection and monitoring electronics.

**Figure 5:**
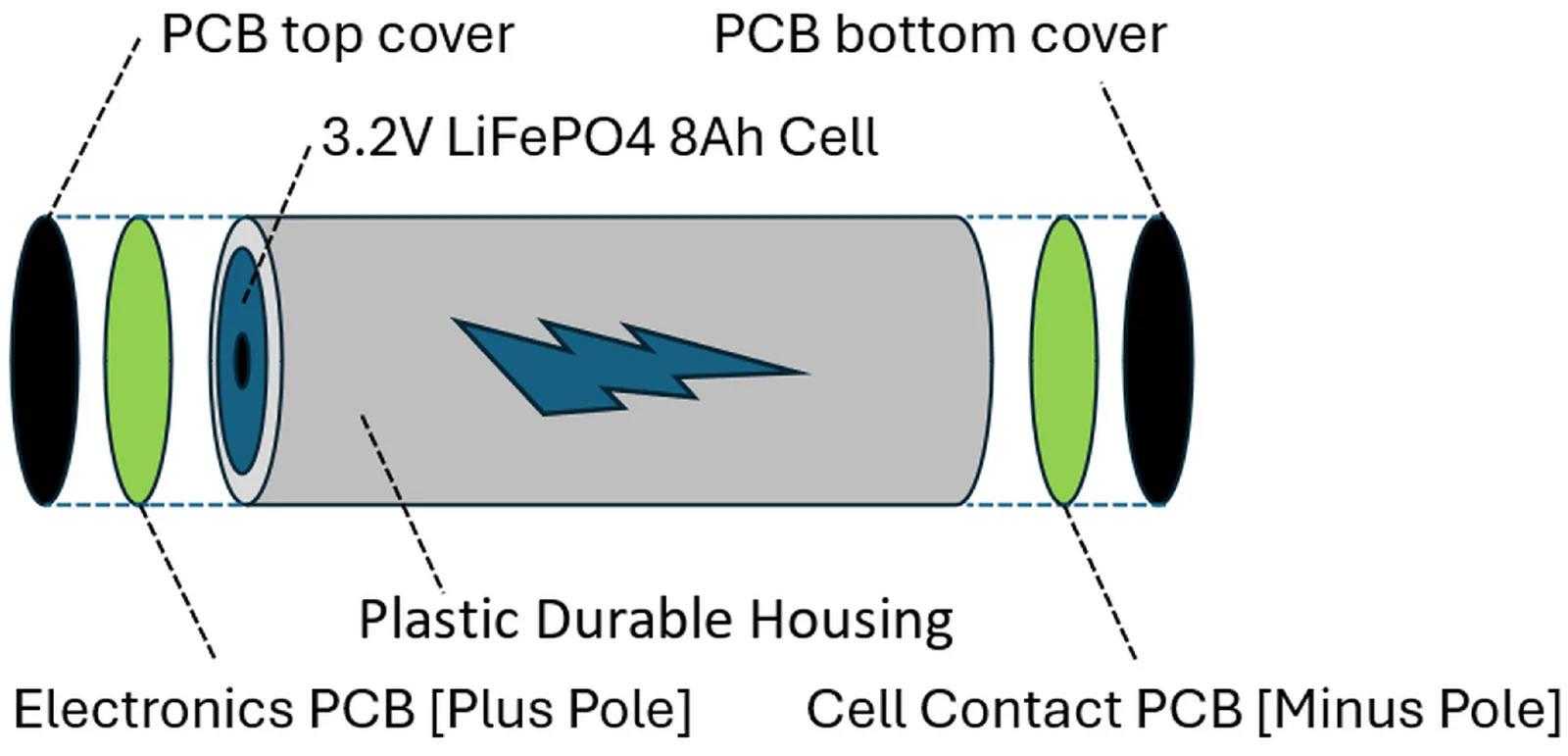
Proposed LiFePO_4_ battery-pack assembly.

The circuit-level implementation is documented in Figure 1, Figure 2, Figure 3, Figure 4 and Figure 5. Figure 1 shows the BQ29729DSET protection circuit, Figure 2 shows the MAX17055ETB+T fuel-gauge circuit, and Figure 3 shows the complete battery-pack interconnection schematic. Figure 4 and Figure 5 show the PCB implementation and assembly arrangement.

## 5. Experimental Setup and Methodology

**Figure 6:**
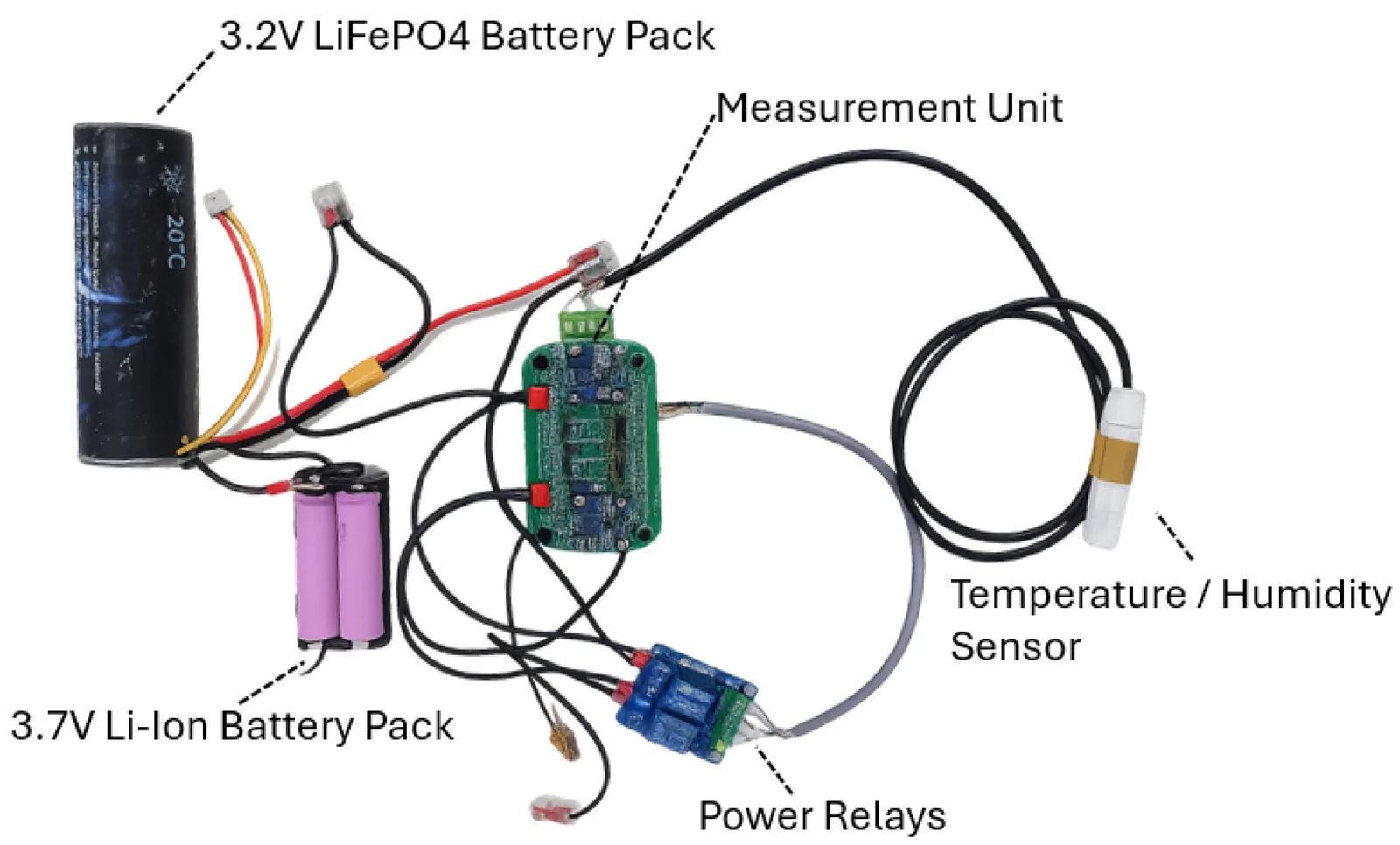
Experimental setup for low-temperature testing of the proposed battery pack.

**Figure 7:**
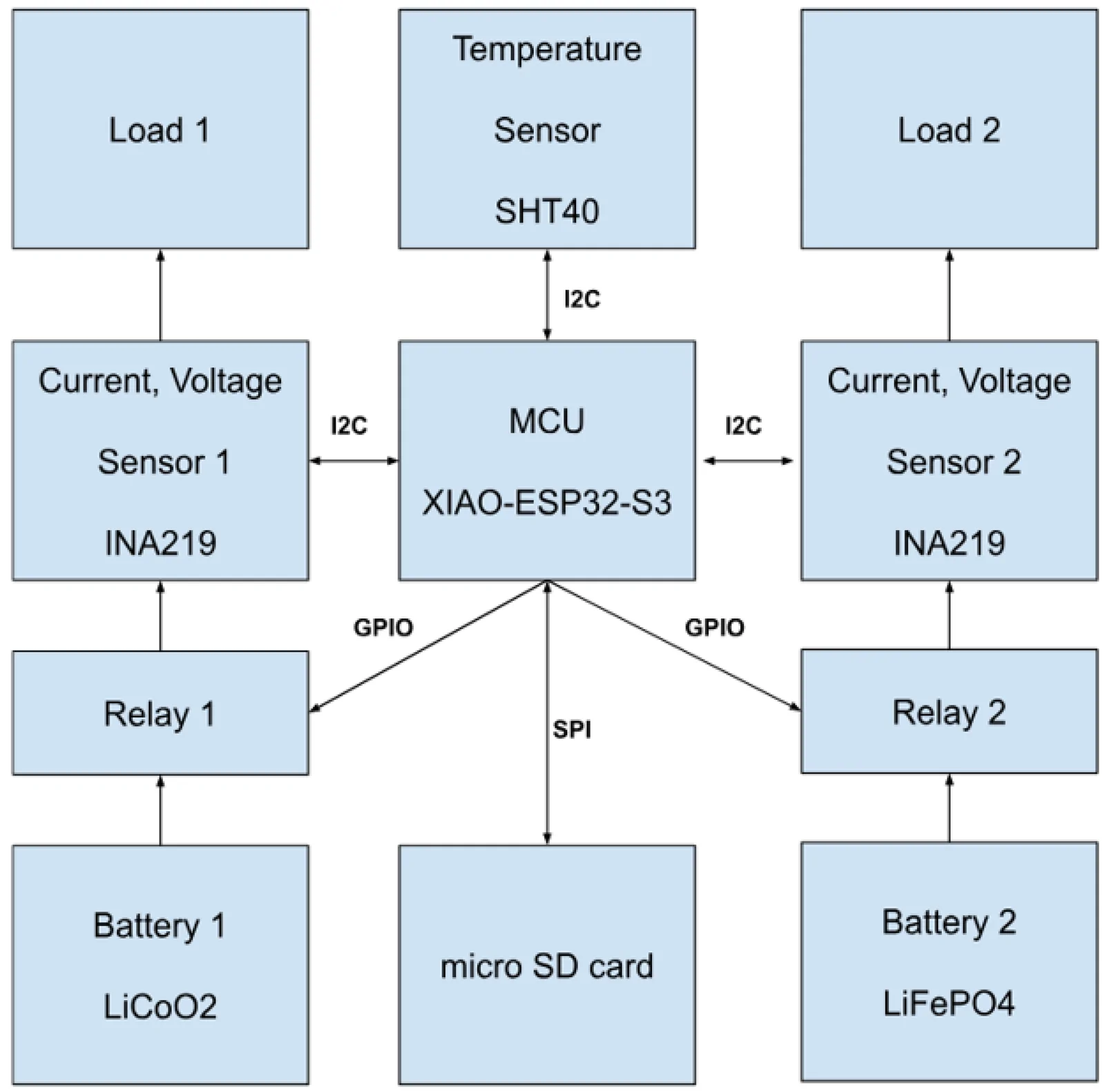
Block diagram of the discharge-measurement platform, including the ESP32-S3 controller, INA219 voltage and current sensors, SHT40 environmental sensor, relay-controlled cutoff, MCP1700/resistor loads, Wi-Fi communication, and microSD storage.

The discharge-measurement platform, shown in Figure 6 and Figure 7, was built around an ESP32-S3 microcontroller (Espressif Systems (Shanghai) Co., Ltd., Shanghai, China). Battery voltage and discharge current were measured using INA219 sensors (Texas Instruments, Dallas, TX, USA) connected through the I^2^C bus. Ambient temperature and relative humidity were recorded using an SHT40 sensor (Sensirion AG, Stäfa, Switzerland). The SHT40 measured chamber-air temperature; it did not directly measure the internal cell temperature.

The discharge load consisted of an MCP1700-2502E/TO low-dropout regulator (Microchip Technology Inc., Chandler, AZ, USA) and selectable power resistors. The equivalent load resistances were approximately 50, 25, and 12.5 Ω, providing nominal load-current conditions of approximately 50, 100, and 200 mA, respectively, at a regulated output of 2.5 V. The constructed load had a maximum intended current capability of approximately 250 mA.

The MCP1700/resistor circuit was not treated as an ideal constant-current electronic load. The regulator maintained approximately 2.5 V across the selected resistor while it remained in regulation, giving $I_{\mathrm{nom}}=2.5/R_{\mathrm{load}}$. The MCP1700 datasheet reports a typical dropout voltage of 178 mV at 250 mA and defines dropout at a 2% output-voltage reduction [23]. The actual current could vary with battery voltage, regulator dropout, resistor tolerance, temperature, and component self-heating. Because complete INA219 current records were unavailable for the analyzed result set, nominal current was used according to Equation (4); the resulting quantities are identified as calculated capacity and calculated energy.

The ESP32-S3 sampled the sensors at 1 Hz and stored values averaged over non-overlapping 30 s intervals. It also controlled relays that disconnected the tested power source when the software-defined cutoff was reached. The cutoff was 2.5 V for the Headway HW 38120HP cell and 2.8 V for the Samsung ICR18650-26H pack. These configuration-specific cutoffs were selected to remain close to the respective manufacturer limits, but their difference prevents a cutoff-identical comparison and is treated as a limitation. Records were transmitted through Wi-Fi and stored locally on a microSD card to reduce the risk of data loss during the long-duration experiments.

The relay-controlled cutoff was part of the measurement platform and depended on correct firmware operation. It was therefore not treated as an autonomous battery-protection function. Host-independent fault-detection hardware was implemented using the BQ29729DSET circuit described in Section 4.2.

The hardware components of the measurement platform are summarized in Table 6.

The tested power-source configurations and the corresponding number of samples and discharge records are summarized in Table 7.

### 5.1. Tested Power-Source Configurations and Sample Size

The experiment compared one Headway HW 38120HP LiFePO_4_ cell with one Samsung ICR18650-26H reference pack consisting of three cells connected in parallel. The three-cell reference pack had a nominal capacity of approximately 7.8 Ah, whereas the HW 38120HP cell had a nominal capacity of 8.0 Ah. The configurations were therefore selected to provide similar nominal capacities.

One complete power-source assembly of each type was tested. The full factorial design comprised two power-source configurations, three ambient-temperature set points, and three nominal currents, yielding 18 discharge runs. One terminal-voltage–elapsed-time plot image was retained for each configuration–temperature–current combination. Repeated measurements using independently assembled packs were not performed; this study is therefore descriptive and does not report inferential statistics or confidence intervals.

The two configurations differed in cell count, geometry, nominal energy, thermal mass, internal resistance, and rated current capability. Because only one assembled configuration of each type was evaluated, the results do not provide statistical confidence intervals and are restricted to the tested assemblies.

### 5.2. Environmental and Load Conditions

Discharge experiments were conducted at nominal ambient temperatures of −30 °C, 8 °C, and 25 °C. At each temperature, nominal discharge currents of 50 mA, 100 mA, and 200 mA were applied, resulting in nine test conditions for each power-source configuration.

Before each experiment, the tested power source was charged using a TD 610 Pro charger (Shenzhen G.T. Hobbies Co., Ltd., Shenzhen, China) with a chemistry-appropriate program. The charged source was then placed in the controlled-temperature chamber and allowed to stabilize for 30 min before the load was connected. The charger current, constant-voltage termination duration, and post-charge rest voltage were not retained in the available experimental record; this limits exact replication of the initial-state conditioning and is acknowledged in Section 8.11.

Each test was terminated when the measured terminal voltage reached the specified software cutoff for that configuration; the relay then disconnected the load. The BQ29729DSET protection circuit was not intentionally triggered during normal discharge testing. No disconnection attributable to the protection circuit was identified in the surviving plot images; this absence is not treated as functional verification.

The −30 °C condition was treated as a severe low-temperature stress test rather than evidence that either manufacturer approves unrestricted operation at that temperature. The SHT40 recorded chamber-air temperature during the original campaign, but the run-level temperature histories and chronological run order were not retained with the surviving discharge-plot images. Consequently, this revision cannot report the mean, range, or sequence for the three anomalous ICR18650-26H runs at −30 °C, and temperature or order effects cannot be excluded. Cell-core temperature and spatial temperature gradients were not measured.

## 6. Data Processing and Evidence Assessment

The voltage–time curves digitized from the surviving plot images were evaluated using runtime, calculated capacity, calculated energy, average discharge voltage, and capacity retention. Runtime, $t_{\mathrm{end}}$, was defined as the elapsed time between load application and the configuration-specific software cutoff. Capacity and energy were calculated with the nominal current according to Equation (4). Capacity retention was calculated with Equation (5). Because only one plot image was retained per condition, the analysis is descriptive; no means across replicates, uncertainty intervals, or significance tests are reported.

The average discharge voltage was calculated as

(7) $${\bar{V}}_{\mathrm{calc}}=\frac{E_{\mathrm{calc},\mathrm{Wh}}}{Q_{\mathrm{calc},\mathrm{Ah}}},$$

where $E_{\mathrm{calc},\mathrm{Wh}}$ is the energy calculated with nominal current in watt-hours, and $Q_{\mathrm{calc},\mathrm{Ah}}$ is the corresponding calculated capacity in ampere-hours.

The voltage–time curves reconstructed by digitizing the surviving plot images were used to identify the cutoff time and to integrate calculated energy. No quantitative initial-voltage-drop or plateau-slope metric is reported because the available images did not support a consistent pre-load reference and measured-current-based capacity axis across all 18 conditions. Voltage-profile comparisons are therefore limited to direct visual interpretation of the curves and are not used to claim statistically demonstrated plateau superiority.

### 6.1. Regulator-Dropout Sensitivity Bound

The dropout assessment uses an explicit approximation rather than treating the missing current as reconstructed data. Over the tested range, the typical 178 mV dropout value at 250 mA was scaled linearly with nominal current:

(8) $$V_{\mathrm{DO},\mathrm{assumed}}\left(I\right)=178\;\mathrm{mV}\frac{I}{250\;\mathrm{mA}},\;V_{\mathrm{loss}}=2.5\;V+V_{\mathrm{DO},\mathrm{assumed}},$$

giving assumed dropout values of 35.6, 71.2, and 142.4 mV and corresponding battery-terminal thresholds of 2.536, 2.571, and 2.642 V at 50, 100, and 200 mA. This is a typical-characteristic sensitivity analysis, not a guaranteed device bound: the datasheet specifies 350 mV as the maximum dropout at its stated full-load test condition, and the actual regulator temperature and unit-specific characteristic were not recorded [23].

**Figure 8:**
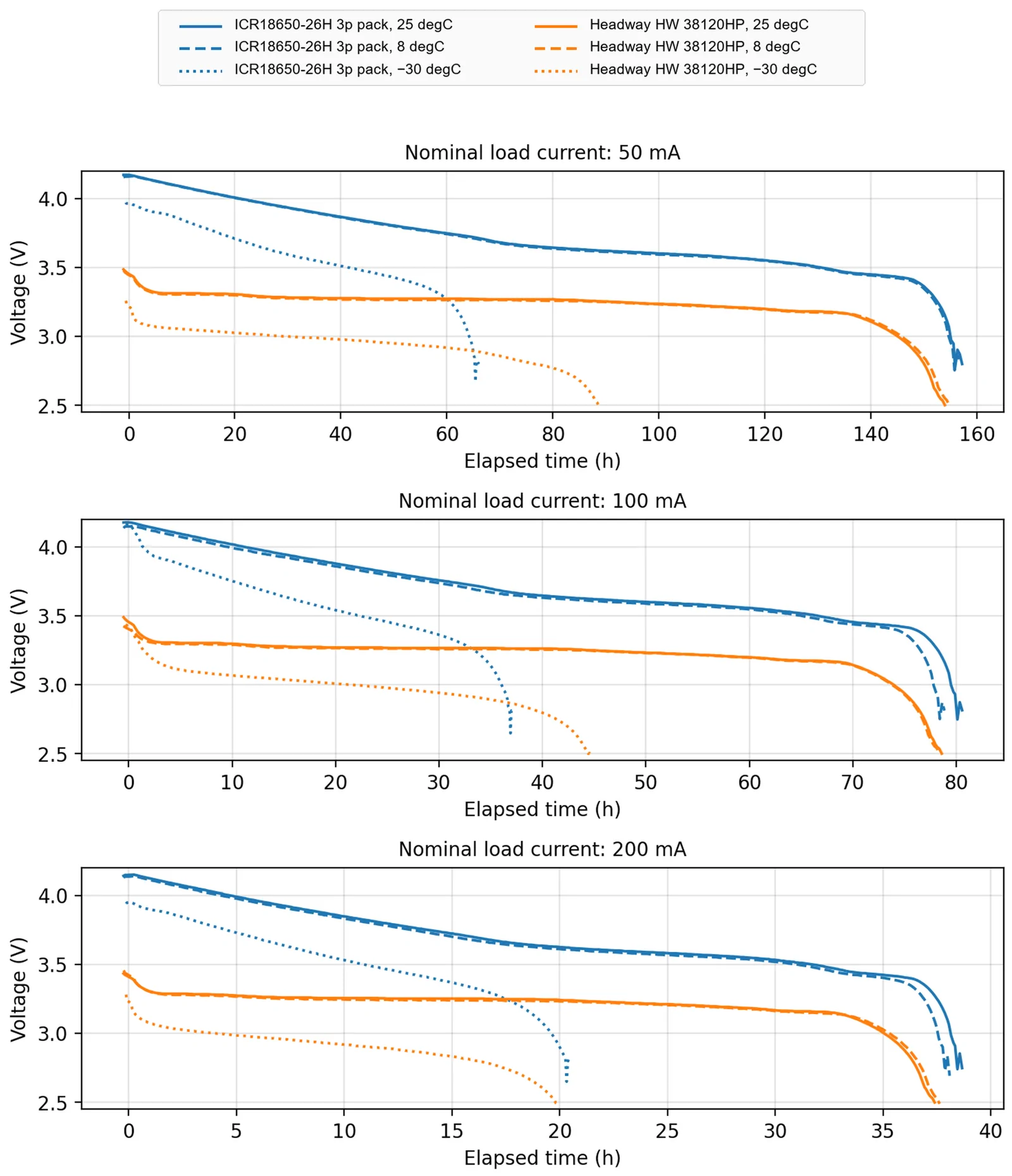
Digitized terminal-voltage curves for the Samsung ICR18650-26H reference pack comprising three parallel cells (blue) and the Headway HW 38120HP cell (orange). Panels correspond to nominal currents of 50, 100, and 200 mA; solid, dashed, and dotted lines denote 25, 8, and −30 °C, respectively. Each curve represents one discharge record and ends at its configuration-specific software cutoff. The vertical segment at the end of a trace denotes measurement termination and does not represent additional measured voltage samples.

At the 2.8 V ICR18650-26H software cutoff, the available headroom above the three assumed regulation-loss thresholds was 264, 229, and 158 mV, respectively. The ICR runs therefore terminated before the assumed dropout region at every nominal setting. By contrast, the 2.5 V HW 38120HP cutoff was below every threshold. The cold HW curves in Figure 8 crossed the estimated thresholds at approximately 88.0, 43.8, and 18.8 h. Relative to their endpoints of 89.1, 44.7, and 20.0 h, the time recorded after the estimated loss of regulation was therefore no more than approximately 1.1, 0.9, and 1.2 h, or 1.2%, 2.0%, and 6.0% of the corresponding runtimes (Table 8). These outward-rounded intervals bound the part of each cold HW record that could be affected under the stated typical-characteristic assumption; they do not recover the current within that interval.

For the two settings used in RQ1, an intentionally conservative sensitivity check subtracts the complete potentially affected HW interval, as if it contributed no comparable regulated-load time. The remaining HW times are 88.0 and 43.8 h, still 22.0 h (33.3%) and 6.6 h (17.7%) longer than the corresponding ICR runtimes. These are lower sensitivity bounds, not corrected runtimes; measured-current integration would be required for a correction.

### 6.2. Analytical and Experimental Evidence Chain

The evidence logic uses two types. First, analytical evidence comprises the component-compatibility assessment, protection-threshold interpretation, fuel-gauge register calculations, Equations (1)–(5), and the terminal-voltage model. Second, experimental evidence comprises the 18 surviving discharge-plot images and the voltage–time curves digitized from them; the communication and storage paths are documented implementation features rather than validated data-redundancy results. No numerical or electrothermal simulation was performed, calibrated, or used to support O1–O3 or RQ1. This boundary is stated explicitly because a simulation without cell-specific impedance, thermal, and current records would not provide independent validation. A calibrated electrothermal model is instead included in the future-work programme in Section 8.

### 6.3. Scope of the Fuel-Gauge Assessment

The present assessment documents the MAX17055ETB+T circuit, configuration values, intended initialization sequence, and register-readback procedure described in Section 4.3. Synchronized fuel-gauge state of charge output and complete measured-current records were not available for the discharge dataset reconstructed from the 18 surviving plot images. Fuel-gauge estimation error, remaining-capacity accuracy, runtime-prediction accuracy, and successful operation of the complete gauge chain are therefore outside the reported experimental results.

### 6.4. Scope of the Protection-Circuit Assessment

The present study documents the implemented BQ29729DSET circuit and assesses the compatibility of its nominal datasheet thresholds with the selected cell and application. Controlled measurements of undervoltage, overvoltage, overcurrent, short-circuit, response delay, and recovery were not performed on the assembled PCB. The results therefore distinguish implementation from experimental verification. In particular, the 4.275 V overvoltage threshold is evaluated only as a secondary abnormal-condition threshold because it exceeds the normal maximum charging voltage of the selected LiFePO_4_ cell. No cell was intentionally overcharged or short-circuited during the discharge experiment.

## 7. Results

The results are organized against O1–O3 and RQ1. O2 and RQ1 are addressed by the discharge-side comparison of the proposed LiFePO_4_ assembly and the Samsung ICR18650-26H reference pack. O1 is addressed by the implementation evidence and verification status reported at the end of this section. O3 requires their joint interpretation and is therefore completed in Section 8. The 18 voltage–time curves digitized from the surviving plot images cover nominal load currents of 50, 100, and 200 mA at ambient-temperature set points of 25, 8, and −30 °C. Figure 8 provides a direct visual comparison; quantitative claims are restricted to the runtime- and nominal-current-based quantities in Table 9.

The discharge curves exhibited an initial voltage decrease following load application, a central discharge region with a comparatively gradual voltage change, and a final steep voltage decrease near the cutoff condition. Differences between the tested configurations were most apparent at −30 °C and near the end of discharge. The electrochemical interpretation of these regions is discussed in Section 8.

### 7.1. Results for O2 and RQ1: Capacity, Energy, and Runtime

At 8 °C and 25 °C, the two configurations produced similar runtimes under each nominal setting. Their calculated capacity estimates were also close, while the ICR18650-26H reference produced higher calculated energy and average voltage. These calculated quantities remain conditional on the nominal-current approximation.

At −30 °C, the proposed assembly ran for 89.1 versus 66.0 h under the 50 mA nominal setting and 44.7 versus 37.2 h under the 100 mA setting. As an answer to RQ1, the observed differences were 23.1 h (35.0%) and 7.5 h (20.2%), respectively, for the two tested source–load configurations. The dropout sensitivity bound reduces these differences to no less than 22.0 h (33.3%) and 6.6 h (17.7%) when the complete potentially affected HW intervals are subtracted. At 200 mA, the runtimes were 20.0 and 20.4 h; this diagnostic observation is not interpreted as evidence of equal delivered capacity.

### 7.2. Anomalous Load Dependence at −30 °C

The ICR18650-26H reference exhibits an anomalous monotonic increase in calculated capacity at −30 °C: 3302, 3721, and 4087 mAh at nominal settings of 50, 100, and 200 mA. The increase is 12.7% from 50 to 100 mA, 9.8% from 100 to 200 mA, and 23.8% across the complete range. Such behaviour is not accepted as a physical increase in accessible capacity with load. It is absent from the warmer ICR records and is not reproduced in the same form by the HW 38120HP assembly.

Self-heating at these low currents is not adopted as an explanation. The asymmetric dropout analysis in Section 6 shows that the ICR cutoff retained 158–264 mV above the assumed regulation-loss voltage; typical-characteristic MCP1700 dropout therefore cannot explain the anomalous ICR trend. The digitized HW curves, not the ICR curves, contain terminal portions below the assumed regulation threshold. In those HW portions, multiplying elapsed time by $I_{\mathrm{nom}}$ can inflate $Q_{\mathrm{calc}}$, with the known sign and upper sensitivity bounds reported in Table 8. Run-level chamber-temperature histories and chronological run order were not retained with the surviving plot images, so temperature variation, charge conditioning, or order effects remain possible explanations for the ICR anomaly.

The complete INA219 records required to separate these mechanisms are unavailable. Therefore, Table 9 preserves the calculated values for transparency but does not treat the −30 °C/200 mA value as a measured capacity or use it to claim similarity between the assemblies. The anomaly also prevents a quantitative claim of capacity-retention superiority from these records alone.

### 7.3. Calculated Capacity Retention

**Figure 9:**
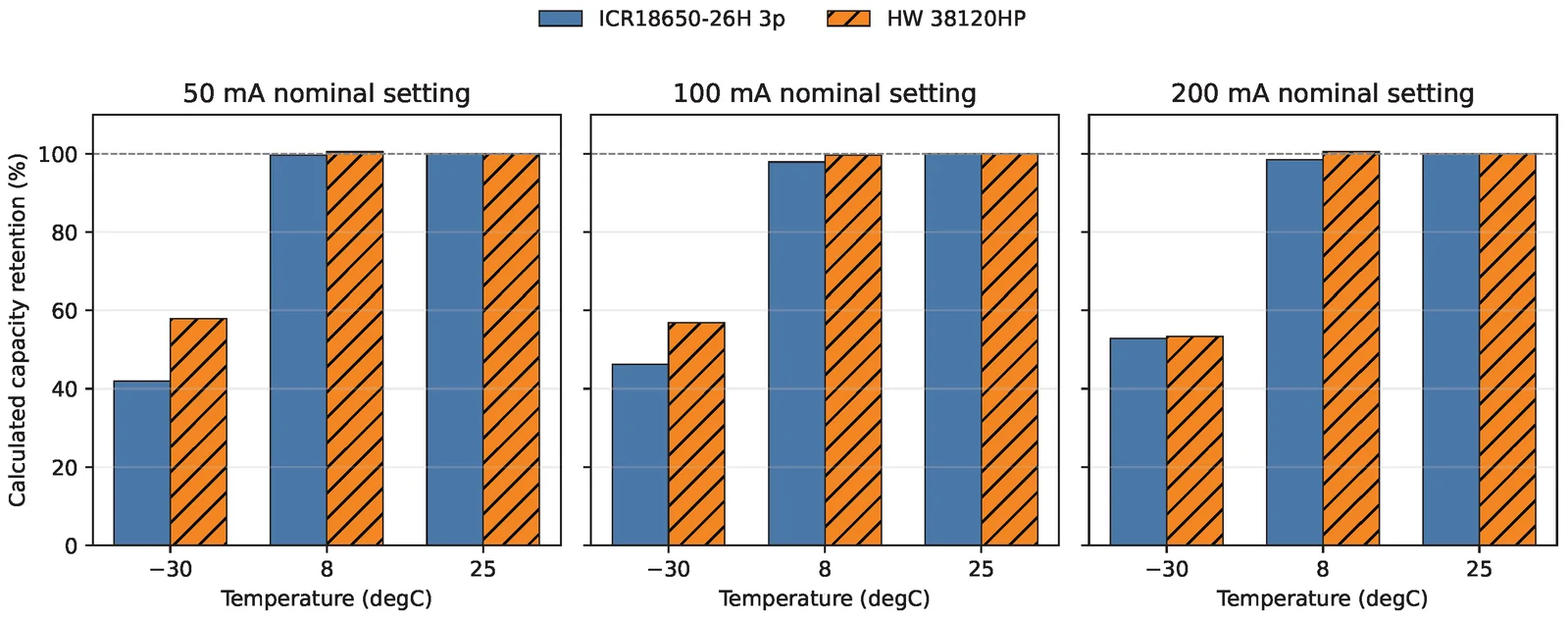
Calculated capacity retention at the three measured ambient-temperature set points, shown as grouped bars to avoid implying interpolation between temperatures. Each panel represents one nominal load setting, and every value is normalized to the corresponding 25 °C record for the same assembly and setting. Each bar is one record; no error bars are shown because replicates were unavailable.

Figure 9 shows that the calculated estimates at 8 °C were close to their room-temperature values. The HW 38120HP values of 100.5% and 100.6% exceed 100% by only 0.5 and 0.6 percentage points. With one record per condition, nominal rather than measured current, and incompletely retained charge-conditioning information, these small excesses are interpreted as run-to-run and calculation variation, not as a demonstrated capacity increase. The cold ICR bars retain the anomalous load dependence described above.

### 7.4. Result for O1: Architecture-Verification Status

The BQ29729DSET protection thresholds reported in this manuscript are the nominal values specified in the component datasheet. Measured undervoltage, overcurrent, short-circuit, and recovery thresholds for the assembled protection PCB were not available for the present analysis. The protection circuit is therefore described as implemented but not fully experimentally verified.

The 4.275 V overvoltage threshold was evaluated only with respect to its compatibility with the selected LiFePO_4_ cell. Because this threshold exceeds the normal maximum charging voltage of the cell, the BQ29729DSET overvoltage function is treated as secondary abnormal-condition protection rather than as the normal chemistry-specific charge-termination mechanism.

Together with the documented MAX17055ETB+T register configuration and host interface, these results satisfy the documentation and implementation criterion of O1. They do not satisfy full functional verification: quantitative protection and fuel-gauge results are absent and remain qualification requirements under O3.

## 8. Discussion

### 8.1. Internal Evaluation of the Objectives and Research Question

Table 10 closes the evidence chain defined in Table 3. It distinguishes analytical evidence from experimental evidence and explicitly records the absence of a numerical simulation. This distinction prevents a circuit calculation or a proposed validation procedure from being presented as an experimentally verified pack function.

The evidential balance is therefore asymmetric. The measured part consists of one voltage–time record per source–temperature–load combination, and even its nominal-current capacity calculation contains an unresolved anomaly. The architecture that motivates the design documentation has no recorded protection trip, recovery, or fuel-gauge output. Physical implementation is not treated as functional validation. RQ1 is answered only by descriptive time-to-cutoff differences under two nominal source–load settings; the absence of current logs, independent assemblies, and repeated tests precludes equal-load, population-level, or chemistry-level inference.

### 8.2. Interpretation of the Discharge-Curve Shape

The discharge curves contain three visually distinguishable regions. The first region is characterized by a voltage decrease following load application. According to Equation (6), this decrease is associated with the instantaneous ohmic term $I\left(t\right)R_{0}\left(T\right)$, contact and interconnection resistance, and short-term electrochemical relaxation.

The central region shows a comparatively gradual voltage change over a substantial part of the discharge. Because the nominal settings were low relative to the rated discharge capability of the cells, several warm-condition curves appear similar through this interval.

The final region is characterized by a steep decrease in terminal voltage near depletion. This behaviour is consistent with increased polarization and effective internal resistance. No plateau-slope or initial-load-drop hypothesis is made: A consistent pre-load reference was unavailable, and the missing current records prevent conversion to a measured delivered-capacity axis. The curve-shape discussion is therefore qualitative and is not used to answer RQ1.

### 8.3. Low-Temperature Performance

The −30 °C condition shortened the time to cutoff for both assemblies relative to their corresponding warm runs. Under the 50 and 100 mA nominal settings, the HW 38120HP assembly ran 35.0% and 20.2% longer than the ICR reference, respectively. These observations answer RQ1 descriptively.

The corresponding retention values of 57.9% versus 42.0% and 56.9% versus 46.2% are calculated from nominal current and time. They are shown for transparency but are not promoted to measured-capacity differences because the anomalous ICR trend demonstrates that current constancy cannot be assumed. The 200 mA calculated-retention similarity is specifically withdrawn as a performance conclusion.

The −30 °C test is interpreted as a severe environmental stress condition. It does not establish that either cell configuration is approved for unrestricted continuous operation at this temperature. Any field deployment must remain within the operating limits specified by the relevant cell manufacturer or must be supported by additional application-specific qualification testing.

### 8.4. Comparisons with Recent Work

Table 11 compares the present results with representative recent studies closest to the thermal, monitoring, and outdoor-IoT functions of the proposed pack. The reported metrics are not interchangeable: Capacity retention measures cold discharge, estimation error measures a monitoring algorithm, and prediction accuracy measures an energy-management service. They are presented together to locate the contribution and its trade-offs, not to construct an artificial overall ranking.

The internal reference comparison supports only a conditional runtime statement. At −30 °C, the proposed assembly ran longer under the 50 and 100 mA nominal settings. At 25 °C, the ICR reference had 17.0–18.9% higher calculated energy, but this too is a nominal-current estimate. The records do not support a general delivered-capacity or net-energy ranking.

Bressan et al. reported approximately 65% retention for a different commercial LiFePO_4_ module near −20 °C [12], and Jia et al. reported 90% capacity output with active CNT heating at −30 °C [13]. The present nominal-current retention estimates are not a direct benchmark against either value. A valid comparison would require calibrated current, matched cutoffs and conditioning, heater consumption, duty cycle, insulation, ambient exposure, and load profile under one protocol.

Relative to the closest IoT-oriented studies, the claimed contribution is not greater energy density, lifetime, or state of charge accuracy. It is the joint documentation of temperature-dependent source behaviour, an independent protection topology, and a host-accessible gauge configuration for the same sensor-node power subsystem. In contrast, Refs. [9,10,11] demonstrate estimation, balancing, remote monitoring, or predictive control capabilities that the present work does not validate. This separation defines a documentation contribution relevant to sensor-system dependability without overstating it as component-level novelty or verified operation.

### 8.5. Practical Implications and Engineering Significance

Under the tested nominal-load conditions, which serve as simplified low-power load cases, the observed time to cutoff was longer for the proposed assembly: 89.1 versus 66.0 h at the 50 mA setting and 44.7 versus 37.2 h at the 100 mA setting. This could extend the interval available for data transmission, controlled shutdown, or maintenance during a cold event. Because actual-current equivalence is unverified, these values must not yet be converted into a field-runtime guarantee. The lower terminal voltage also requires explicit checking of the downstream converter, radio-current peaks, and brownout threshold.

The sensor-oriented engineering rationale is to treat the power source as an observable subsystem with host-independent protection hardware rather than as an interchangeable cell holder. Host-accessible battery-state information can support adaptive sensing and communication policies, while the protection architecture is designed to operate independently of the host firmware. Realizing those operational benefits depends on measured gauge accuracy and protection behaviour, which the present dataset does not establish.

Implementation in a harsh outdoor enclosure introduces further challenges: condensation and ingress control, connector and PCB resistance, cell-temperature gradients, cold-charge inhibition, solar-charger compatibility, converter efficiency over the complete voltage range, radio pulse loads, and communication loss during low-voltage events. Passive insulation may reduce exposure but can also retain heat during warm operation; active heating can recover cold capacity but consumes stored energy and adds control-failure modes. These choices must therefore be evaluated at the complete sensor-node level rather than from cell capacity alone.

### 8.6. Future Research Directions

The immediate validation programme is to repeat every condition with multiple independently assembled packs and complete calibrated current logs; apply realistic sleep–sense–compute–transmit pulse profiles; measure cell-surface and chamber-air temperatures; and quantify uncertainty in capacity, energy, and retention. Controlled fault injection should then determine protection trip points, delays, and recovery, while synchronized coulomb counting should quantify MAX17055 state of charge, remaining-capacity, and runtime error against benchmarks such as Ref. [9].

A cell-specific electrothermal model can be identified only after measured current, impedance, and temperature data are available. Such a model should be calibrated on a subset of the experiments and prospectively validated on withheld temperature–load conditions; it should then compare passive insulation, duty-cycled heating, and unheated operation on a net-energy basis. Finally, multi-season outdoor trials should evaluate ageing, self-discharge, enclosure effects, charging below zero, energy-harvesting interaction, telemetry availability, and maintenance interval. These steps would convert the present feasibility evidence into the field-reliability evidence required for a harsh-environment IoT sensor product.

### 8.7. Protection-Architecture Implications

The architecture implements BQ29729DSET hardware intended for fault detection independently of the host microcontroller. Its datasheet-specified 2.300 V undervoltage threshold, current-fault thresholds, and short-circuit response are relevant to the independent protection concept. However, the actual trip currents depend on the resistance of the external MOSFETs, PCB traces, and interconnections and cannot be determined solely from the voltage thresholds listed in the component datasheet.

The 4.275 V overvoltage threshold is above the normal maximum charging voltage of the selected LiFePO_4_ cell. BQ29729DSET therefore cannot provide normal chemistry-specific charge termination for this cell. Its overvoltage function is interpreted as secondary protection against an abnormal condition, while normal charge-voltage regulation must be provided by a separate charging system.

Because measured protection thresholds, response delays, and recovery behaviour were not available, the protection circuit cannot yet be described as experimentally verified. Controlled testing of the assembled PCB is required before the pack is claimed to provide fully validated field protection.

### 8.8. Fuel-Gauge Integration

MAX17055ETB+T was configured for the 8 Ah HW 38120HP cell using a 10 mΩ sense resistor, DesignCap = 0x3E80, VEmpty = 0x964E, IChgTerm = 0x0A00, and the standard ModelID = 6 LiFePO_4_ model. These settings establish a documented and reproducible fuel-gauge configuration.

However, configuration correctness does not by itself demonstrate state of charge accuracy. Quantitative validation requires comparisons of the MAX17055ETB+T output with a reference obtained from measured current integration, as described in Section 6. Because corresponding fuel-gauge records were not available for the present result set, no numerical claim is made regarding state of charge error, remaining-capacity accuracy, or runtime-prediction accuracy.

The use of the standard ModelID = 6 model instead of a cell-specific characterized model may also limit accuracy, particularly because the relatively flat open-circuit-voltage profile of LiFePO_4_ cells makes voltage-only state estimation difficult over the central state of charge interval.

### 8.9. Measurement-Load Limitation

The MCP1700/resistor discharge circuit does not behave as an ideal constant-current electronic load over the complete battery-voltage and temperature ranges. The actual current may vary because of regulator dropout, resistor tolerance, temperature dependence, and component self-heating. The typical-characteristic analysis places the ICR cutoffs above the assumed dropout thresholds and bounds the potentially affected terminal intervals of the cold HW records at approximately 1.1, 0.9, and 1.2 h for the three nominal settings.

Complete INA219 current records were not available. The reported capacity and energy values are therefore consistently called calculated values and use nominal current. The monotonic increase from 3302 to 4087 mAh shows that the nominal-current-based estimates are condition-dependent in the digitized cold ICR curves. The available data do not identify a unique cause; the dropout sensitivity calculation rules out typical-characteristic MCP1700 dropout as the explanation for this ICR anomaly but does not distinguish the remaining temperature, conditioning, or run-order mechanisms.

The data support a preliminary comparison of measured time to the configuration-specific cutoff under nominal load settings. Logger-derived current integration is required before capacity, energy, current stability, or equal-load performance can be claimed quantitatively.

### 8.10. Assessment Against Application Requirements

The results can be compared with the application requirements and selected implementation parameters in Table 4. The surviving plot images and the curves digitized from them document discharge time and voltage at the specified temperature and nominal-load settings. The hardware design includes Wi-Fi and microSD paths, but a formal record-by-record verification of their redundancy was not retained.

The discharge experiment evaluated the two source assemblies and the measurement load; it did not exercise the BQ29729DSET fault states or record MAX17055 estimates. Accordingly, no protection or monitoring function is marked as validated. Measured protection thresholds, fuel-gauge error, current stability, and long-term field behaviour remain unresolved.

External consistency was assessed by comparing the implemented cell and IC parameters with the corresponding manufacturer specifications for the Samsung ICR18650-26H, Headway HW 38120HP, BQ29729DSET, and MAX17055ETB+T [19,20,21,22]. This comparison confirms the nominal component parameters but does not replace experimental verification of the assembled system.

### 8.11. Limitations

Only one assembled configuration of each power-source type was evaluated, and one discharge record was available for each temperature–current condition. Statistical confidence intervals and assessment of cell-to-cell or pack-to-pack variation are therefore not available.

The two configurations were matched approximately by nominal capacity but differed in nominal energy, cell count, geometry, thermal mass, internal resistance, and rated current capability. The measured differences consequently reflect the complete power-source constructions rather than battery chemistry alone.

Capacity and energy were calculated using nominal current because complete current logs were unavailable. The anomalous increase in the calculated ICR capacity with nominal current at −30 °C shows that this approximation is condition-dependent and invalidates a delivered-capacity comparison at 200 mA. The voltage curves were digitized from the surviving plot images; the original machine-readable voltage–time series are unavailable, and digitization and time-base uncertainty were not independently quantified. The dropout bound uses a linearly scaled typical datasheet characteristic rather than a unit- and temperature-specific measured characteristic; it is therefore a sensitivity bound, not a reconstructed current trace. The chemistry-specific charger program was identified, but the detailed charge current, termination duration, and post-charge rest voltage were not retained. Run-level chamber-temperature histories and the chronological run order were also not retained. The SHT40 measured chamber air rather than cell-core temperature, and a 30 min stabilization interval does not prove that the two assemblies reached identical internal temperatures.

MAX17055ETB+T’s fuel-gauge output was not quantitatively validated against a coulomb-counted reference, and BQ29729DSET’s thresholds, delays, and recovery behaviour were not measured on the assembled PCB. The protection and monitoring circuits are consequently described as implemented and configured, respectively, rather than fully validated.

This study addresses discharge behaviour, protection architecture, and monitoring integration. Charging-circuit design, charge-side validation, ageing, repeated cycling, self-discharge, real IoT duty cycles, and long-term outdoor field performance remain outside its scope.

## 9. Conclusions

O1 was achieved only as design documentation for a compact sensor-node power architecture containing a Headway HW 38120HP cell, BQ29729DSET protection hardware, and a configured MAX17055ETB+T fuel gauge. O2 was achieved as a transparent description of 18 surviving discharge-plot images and the voltage–time curves digitized from them, including their measurement anomaly. O3 was achieved as an evidence and validation-gap audit, not as functional or field qualification.

At −30 °C, the proposed assembly ran for 89.1 versus 66.0 h under the 50 mA nominal setting and 44.7 versus 37.2 h under the 100 mA setting. A typical-characteristic dropout analysis places no ICR record in the assumed dropout region and bounds the terminal portions of the corresponding HW records at approximately 1.1 and 0.9 h. Even subtracting those complete intervals leaves positive runtime differences of 22.0 h (33.3%) and 6.6 h (17.7%). These descriptive sensitivity bounds answer RQ1 for the two source–load configurations; they do not establish equal measured current, chemistry superiority, or statistical significance.

The cold ICR calculated capacity increased anomalously from 3302 to 4087 mAh as the nominal setting increased from 50 to 200 mA. Because the ICR cutoff remained above the assumed dropout thresholds, regulator dropout does not explain this anomaly under the stated typical-characteristic model. Missing current logs, run-level chamber histories, and test order prevent the remaining run-condition effects from being separated. The calculated-retention similarity at 200 mA is therefore not a supported performance conclusion, and no delivered-capacity advantage is claimed.

The contribution relevant to *Sensors* is reproducible documentation of a sensor-node power-subsystem design together with preliminary environmental runtime records and an explicit audit of what they do not validate. The BQ29729DSET threshold mismatch also identifies a required redesign toward chemistry-matched primary protection. MAX17055 accuracy and all protection trip points, delays, and recovery behaviour remain unmeasured. Replicated packs, calibrated current integration, retained temperature histories, controlled fault tests, gauge validation, realistic pulsed loads, ageing, and multi-season outdoor deployment are required before the design can be described as a monitored, protected, or field-qualified harsh-environment battery pack.

## Figures and Tables

**Table 1:** Structured comparison of the representative recent literature and the system-level scope of the present work.

Study	Primary Scope	Pack	Prot.	Mon.	Cold Test	Boundary Relative to This Work
Hasan et al. (2023) [1]	Battery-technology survey and IoT suitability matrix	–	–	–	–	Selection guidance; no implemented pack
Kuaban et al. (2023) [2]	Stochastic IoT battery- depletion modelling	–	–	–	–	Lifetime model; no pack hardware or thermal test
Kim et al. (2024); Park et al. (2025) [3,4]	Communication-layer reduction of IoT battery drain	–	–	–	–	Reduces demand; storage hardware not evaluated
Refs. [5,6,7,8]	Energy harvesting, storage, and energy-aware IoT operation	Part.	–	Part.	–	Focus on harvested-energy availability and scheduling
Pimentel et al. (2025) [9]	Data-driven real-time state of charge estimation	–	–	Yes	–	Estimator validation; no outdoor pack implementation
Parthasarathi et al. (2023) [10]	IoT-connected pack monitoring and passive balancing	Yes	Part.	Yes	–	Fixed-temperature setting
Zurita Macias and Trilles (2024) [11]	Weather-based battery-level prediction for solar IoT nodes	Part.	–	Yes	Ind.	Pack protection not validated
Bressan et al. (2025) [12]	Commercial LiFePO_4_ module at −20 to +55 °C	Yes	–	Part.	Yes	No IoT-oriented protection integration
Jia et al. (2025) [13]	CNT-heated Li-ion pack with sensor feedback at −30 °C	Yes	–	Part.	Yes	Active-heating hardware and energy overhead
**Present work**	**Integrated outdoor-IoT pack and discharge comparison**	**Yes**	**Impl.**	**Config.**	**Yes**	Trip points and gauge accuracy not measured

**Table 2:** Manufacturer-specified characteristics of the two tested power-source configurations.

Parameter	ICR18650-26H Reference Pack	HW 38120HP Configuration
Battery type	Rechargeable Li-ion	LiFePO_4_
Configuration	Three ICR18650-26H cells connected in parallel	One HW 38120HP cell
Number of cells	3	1
Nominal voltage	3.63 V	3.2 V
Nominal capacity	7.8 Ah	8.0 Ah
Nominal energy	28.3 Wh	25.6 Wh
End-of-discharge voltage	2.75 V	Approximately 2.5 V
Maximum charging voltage	4.2 V	3.65±0.05 V
Physical configuration	Three cylindrical 18650 cells connected in parallel	One cylindrical 38120 cell

**Table 3:** Alignment of the research objectives and descriptive research question with the evaluation method and interpretation criterion.

Item	Question	Method and Evidence	Interpretation Criterion
O1	Can the protection and monitoring hardware be documented as one sensor-node power architecture?	Schematic, component-datasheet, threshold-compatibility, register-configuration, and firmware-interface analysis	Architecture and configuration are reproducibly documented; no functional- verification claim is made
O2	What behaviour is present in the 18 surviving discharge-plot images and the curves digitized from them?	Full 2 × 3 × 3 design; time to cutoff, calculated capacity and energy estimates, average voltage, and retention	All digitized curves and the anomalous −30 °C load dependence are reported; nominal-current estimates are not treated as measured capacity
O3	What evidence is usable, and what prevents functional or field qualification?	Joint evaluation of O1, O2, RQ1, requirements, state of the art, and missing records	Supported observations are separated from unresolved protection, gauge, load-current, thermal, ageing, and field tests
RQ1	How does time to cutoff differ at −30 °C and the 50–100 mA settings?	Direct descriptive comparison of recorded runtime under the same nominal load setting	Differences are reported for the two source– load configurations without hypothesis testing or statistical generalization

**Table 4:** Application requirements, selected implementation or test parameters, and evidence available for the IoT battery-pack prototype. Fixed component properties are not presented as design requirements.

Category	Application Requirement	Selected Implementation or Test Parameter	Assessment in the Present Study
Power source	Rechargeable source with documented low-temperature behaviour	Headway HW 38120HP (LiFePO_4_)	Datasheet consistency check
	Compatible source voltage	3.2 V	Terminal-voltage recording during discharge
Operating conditions	Cold, cool, and room- temperature evaluation	−30 °C, 8 °C, 25 °C	Controlled chamber set points; run-level SHT40 histories not retained
	Representative low-power and diagnostic load settings	50 mA, 100 mA, 200 mA	Nominal MCP1700/resistor load setting; complete current logs unavailable
Protection	Host-independent excessive-discharge fault detection	BQ29729DSET fixed undervoltage threshold: 2.300 V	Circuit implementation and datasheet-threshold analysis; trip point not measured
	Secondary abnormal- overvoltage detection	BQ29729DSET fixed overvoltage threshold: 4.275 V; not a LiFePO_4_ design target	Compatibility analysis only; trip point not measured
	Host-independent current- fault detection	BQ29729DSET fixed voltage thresholds	Circuit implementation only; controlled fault injection not performed
Monitoring	Host-accessible battery-state estimate	MAX17055 fuel gauge	Register configuration documented; estimation accuracy not quantified
Data acquisition	Local record backup	microSD storage	Wi-Fi and microSD paths implemented; outputs not formally cross-checked

**Table 5:** Technical constraints considered in the prototype design and interpretation of the experimental results.

Technical Constraint	Implication	Treatment in This Work
BQ29729DSET voltage thresholds	The 4.275 V hardware overvoltage threshold is above the approximately 3.65 V maximum charging voltage of the selected LiFePO_4_ cell.	The BQ29729DSET is used as a secondary hardware fault-protection layer and not as the normal LiFePO_4_ charge-termination mechanism.
Firmware-controlled test cutoff	The relay used to terminate the laboratory discharge tests depends on the measurement-system firmware and is not equivalent to autonomous pack-level protection.	The test-bench cutoff and the BQ29729DSET protection functions are described separately.
Regulated discharge load	The MCP1700 regulates the voltage across the selected load resistor, producing an approximately constant current while the regulator remains outside dropout.	The load current is determined from the regulated output voltage and resistor value. Possible deviations caused by component tolerance, temperature, and dropout near the end of discharge are considered.
Different power-source constructions	The two configurations have similar nominal capacities but differ in cell count, nominal energy, geometry, thermal mass, internal resistance, and rated current capability.	The conclusions are restricted to the two assembled power-source configurations evaluated in the experiments.
Low-temperature stress condition	The −30 °C condition may be outside the manufacturer-specified normal discharge range of one or both tested cell types.	This condition is treated as a severe environmental stress test and not as evidence of unrestricted manufacturer-approved operation.
Limited sample size	One discharge record per configuration and test condition does not support statistical confidence intervals or broad generalization across manufacturers, production batches, or cell formats.	This study is presented as a prototype evaluation. Replicated testing with independently assembled packs is required for statistical comparison.
Incomplete component-level verification	Datasheet values alone do not demonstrate the actual trip thresholds, response delays, and recovery behaviour of the assembled protection circuit.	The measured protection thresholds and recovery behaviour must be established before the pack is described as fully validated for field deployment.

**Table 6:** Measurement-platform components.

Component	Role	Measured or Controlled Quantity
ESP32-S3	Main controller and data logger	Timing, relay control, Wi-Fi upload, and microSD-card logging
INA219	Current and voltage monitor	Battery terminal voltage and discharge current
SHT40	Environmental sensor	Ambient temperature and relative humidity
MCP1700-2502E/TO with selectable resistors	Regulated discharge load	Approximately 50 mA, 100 mA, and 200 mA load conditions
Relay module	Measurement-system cutoff	Load disconnection at the software-defined cutoff voltage
MicroSD card	Local data backup	Timestamped measurement records

**Table 7:** Tested power-source configurations used in the discharge experiment.

Battery Type	Cell or Pack	Nominal Capacity	Samples and Repetitions	Reference
Rechargeable Li-ion	Samsung ICR18650-26H reference pack, three cells in parallel	2600 mAh per cell; approximately 7.8 Ah for the complete parallel pack	One assembled pack and one discharge record per test condition	[19]
LiFePO_4_	Headway HW 38120HP single-cell configuration	8.0 Ah nominal	One cell and one discharge record per test condition	[20]

**Table 8:** MCP1700 dropout sensitivity analysis. Thresholds use Equation (8); cold-HW crossing times are read from voltage–time curves digitized from the surviving plot images.

Nominal *I*	$R_{\mathrm{load}}$	Assumed $V_{\mathrm{DO}}$	$V_{\mathrm{loss}}$	ICR Margin at Cutoff	Maximum Affected Cold-HW Interval
(mA)	(Ω)	(mV)	(V)	(mV)	(h; % of Runtime)
50	50.0	35.6	2.536	264	1.1; 1.2%
100	25.0	71.2	2.571	229	0.9; 2.0%
200	12.5	142.4	2.642	158	1.2; 6.0%

**Table 9:** Descriptive discharge results for the two tested assemblies. Capacity and energy are nominal-current estimates, not measured-current integrals; average voltage is $E_{\mathrm{calc}}/Q_{\mathrm{calc}}$. Retention is normalized to the corresponding 25 °C record for the same assembly and nominal setting. Each row represents one discharge record (n=1).

Cell	Temp.	Nominal *I*	Runtime	Calc. *Q*	Calc. *E*	Avg. *V*	Retention
	(°C)	(mA)	(h)	(mAh)	(Wh)	(V)	(%)
ICR18650-26H 3p	−30	50	66.0	3302	11.78	3.568	42.0
ICR18650-26H 3p	−30	100	37.2	3721	13.27	3.566	46.2
ICR18650-26H 3p	−30	200	20.4	4087	14.37	3.516	52.8
ICR18650-26H 3p	8	50	156.6	7831	28.92	3.693	99.7
ICR18650-26H 3p	8	100	78.9	7886	29.06	3.685	97.9
ICR18650-26H 3p	8	200	38.1	7617	27.95	3.669	98.5
ICR18650-26H 3p	25	50	157.2	7858	29.04	3.696	100.0
ICR18650-26H 3p	25	100	80.6	8057	29.77	3.695	100.0
ICR18650-26H 3p	25	200	38.7	7733	28.46	3.680	100.0
HW 38120HP	−30	50	89.1	4455	13.08	2.936	57.9
HW 38120HP	−30	100	44.7	4468	13.30	2.977	56.9
HW 38120HP	−30	200	20.0	3994	11.57	2.897	53.4
HW 38120HP	8	50	154.7	7736	24.89	3.217	100.5
HW 38120HP	8	100	78.3	7834	25.17	3.213	99.7
HW 38120HP	8	200	37.6	7524	24.03	3.194	100.6
HW 38120HP	25	50	153.9	7696	24.81	3.224	100.0
HW 38120HP	25	100	78.6	7860	25.28	3.216	100.0
HW 38120HP	25	200	37.4	7478	23.94	3.201	100.0

**Table 10:** Internal evaluation of the research objectives and RQ1 using the evidence reported in this study.

Item	Analytical Evidence	Simulation Evidence	Experimental Evidence	Assessment
O1	Threshold compatibility, schematic, sense-resistor scaling, and gauge- register configuration	Not performed or claimed	Assembled hardware is documented; no protection-response or gauge-output record is reported	Achieved only as design documentation; monitoring and protection functions are unverified
O2	Common metric definitions and explicit nominal-current approximation	Not performed or claimed	Eighteen runtime and voltage records; anomalous cold ICR load dependence disclosed	Achieved descriptively; calculated capacity and energy are not validated measurements
O3	Requirements, cutoff hierarchy, anomaly, and state-of-the-art boundary analysis	Not performed or claimed	Runtime evidence plus explicit missing- data inventory	Achieved as an evidence audit, not as functional or field qualification
RQ1	Descriptive runtime difference at both target settings	Not performed or claimed	+23.1 h at 50 mA and +7.5 h at 100 mA	Answered for the two source–load configurations; actual-current equivalence and inference are unavailable

**Table 11:** Quantitative benchmarking against representative recent battery-pack, battery-monitoring, and outdoor-IoT studies. Values use the definitions reported by each source and are not normalized across studies.

Study	System and Condition	Reported Quantitative Result	Relation and Trade-Off
Bressan et al. (2025) [12]	23 Ah commercial LiFePO_4_ module, 0.5C, approximately −10 and −20 °C	Capacity reductions of approximately 15% and 35%, corresponding to approximately 85% and 65% retention	Higher retention at less severe temperatures; different module, C-rate, cutoff, conditioning, and calculation prevent a direct advantage claim
Jia et al. (2025) [13]	CNT-heated Li-ion pack, 2.5 A discharge at −30 °C	Heating to 0 °C in 141 s; 90% capacity and 73% energy output; unheated discharge lasted 45 s	Demonstrates the benefit of active heating within that system, but adds heater mass, feedback control, cycling, and an energy overhead
Parthasarathi et al. (2023) [10]	Four 3.7 V, 1200 mAh cells with passive balancing and cloud monitoring	Four-cell monitored prototype evaluated under fixed environmental- temperature conditions	Adds cell balancing and remote monitoring; does not report the controlled −30 °C source comparison provided here
Pimentel et al. (2025) [9]	Data-driven real-time Li-ion state of charge estimation over ten drive cycles	MAE as low as 0.40%; embedded inference time 1.3–2.2 s	Establishes a quantitative monitoring benchmark; the present MAX17055 configuration has not yet been accuracy-tested
Zurita Macias and Trilles (2024) [11]	Weather-assisted battery-level prediction for outdoor solar IoT nodes	Average prediction accuracy up to 94.09% in selected scenarios	Enables adaptive sampling; does not validate low-temperature pack protection or discharge retention
**Present work**	**Passive HW 38120HP assembly, −30 °C, 50–200 mA nominal settings**	**89.1 versus 66.0 h at 50 mA; 44.7 versus 37.2 h at 100 mA; calculated retention 57.9% at 50 mA and 56.9% at 100 mA; the 200 mA value is not used for performance interpretation**	**Direct runtime evidence but no measured-current capacity; unresolved ICR anomaly and unverified gauge/** **protection functions**

## Data Availability

The surviving experimental material comprises 18 images of individual terminal-voltage–elapsed-time plots, one for each tested configuration–temperature–load condition. Figure 8 and Table 9 were reconstructed from these images by digitization. The plot images can be made available by the corresponding author upon reasonable request. The original machine-readable voltage–time series and CSV files, complete INA219 current logs, run-level chamber-temperature histories, chronological run order, and detailed charging records are unavailable and therefore cannot be shared.
